# Infectious Serology in Total Laboratory Automation: Fit or Unfit

**DOI:** 10.7759/cureus.100217

**Published:** 2025-12-27

**Authors:** Suneeta Meena

**Affiliations:** 1 Infectious Disease, All India Institute of Medical Sciences, New Delhi, IND

**Keywords:** clia, elisa, infectious serology, quality control, standardization, total laboratory automation

## Abstract

Several parameters of infectious serology are increasingly being incorporated into workflows of total laboratory automation (TLA). Infectious serology is not a well-defined field, with each parameter having its own requirement for reporting. The unavailability of uniform international standards further complicates quality control (QC). Infectious serology in total laboratory automation offers opportunities like integration of laboratory services, auto-verification, re-run, and reflex add-on testing. But questionable application of standard QC procedures like Westgard rules, unavailability of international standards, biological variability of pathogens, and host immune response remain challenges that require urgent solutions.

## Editorial

Automation is increasingly being incorporated into laboratory workflows ranging from bundled closed automated to total laboratory automation (TLA). In TLA analysers, performing different types of tests on different sample matrices is physically connected by tracks or belts. Tests vary from clinical chemistry, immunochemistry to haematology. Infectious serology for several blood-borne pathogens is increasingly becoming part of the scope of services offered by these laboratories. Reporting of infectious serology parameters requires not only detailed knowledge of each pathogen in question but also compliance with national and international guidelines. Unlike many quantitative clinical chemistry tests, infectious serology commonly produces qualitative or semi-quantitative results and frequently relies on non-commutable control materials. These features challenge conventional quality control (QC) paradigms and demand adapted QC strategies that preserve clinical relevance and patient safety. In TLA, reporting of infectious serology parameters has to be modified as per the laboratory’s requirements. Also, both national and international guidelines have to be adhered to for reporting of infectious serology parameters [1].

With this backdrop, this editorial summarises current practices, opportunities and challenges associated with reporting of infectious disease parameters in TLA.

Opportunities

The essence of TLA is that different sample matrices can be integrated where different analysers are interconnected by a track-based system. Immunoassay platforms are an integral part of these laboratories. They can be used for testing both immunochemistry and infectious serology parameters. So, from the patients’ perspective, a single sample can be used for a range of tests. There are several advantages of TLA, like increased throughput, improved quality, and reduced turnaround time (TAT), among many others, which are beyond the scope of this review. But for reporting of infectious serology results primary advantages being offered are auto verification, integration, lower sample volume, rerun, reflex, add-on testing, and worker safety are primary.

Samples for screening of blood-borne viruses, especially Hepatitis B virus (HBV), Hepatitis C virus (HCV), and Human Immunodeficiency virus (HIV), are usually received in bulk in laboratories; at times, clinical indication may not be available. So, these laboratories can be used for screening. Middleware of TLA enables efficient integration of test results, thus allowing a broader overview of the patients’ picture. Accordingly, several interventions can be made at the laboratory level, including delta check, retesting, and add-on testing, as the case may be. The requirement for clinical chemistry and serology from a single serum sample makes these laboratories patient-friendly. This feature also makes these laboratories environmentally friendly by reducing the biological waste generated.

Challenges

Infectious serology parameters are mostly qualitative and not quantitative like biochemistry parameters. Even when antibodies are quantified, the binding capacities to the antigen present in the kit are biologically variable. The test systems compare the intensity of a chemical reaction against a predetermined cut-off, giving a signal/cut-off ratio (S/CO). The dose response curve is linear, but it is not indicative of the number of antibodies present in the sample, but intensity of binding. Intensity may decrease or increase with time, depending on the type of antibody detected. Detection of binding antibodies is not equivalent to neutralising antibodies. The performance of commercial assays depends on antigen composition and the methodology of the assay. Assay manufacturers design serological assays to separate positive and negative populations as widely as possible so that very few false positives and false negatives fall close to the cut-off.

The principle of quality control in biochemistry does not apply to infectious serology. The requirement in the latter is to report the result as positive and negative. The concept of total allowable error does not apply in infectious serology. QC values rarely exceed the set limits; even if they do, they do not affect the results. Modification of traditional QC principles for infectious serology parameters has been published, but their application in a laboratory setting is unresolved. Application of Westgard rules introduces an unacceptable level of false rejections, leading to unnecessary re-runs and troubleshooting [2].

Unlike many biochemistry analytes, where international reference materials exist, serology assays frequently use proprietary antigens and differing calibration strategies. International standards are available for a few like rubella, SARS-COV-2, HBV and Measles. But these are also periodically revised as there are various factors influencing commutability of standards like purification, inactivation, sample matrix, i.e., plasma or serum, molecular and antigenic variants of the analyte, subgenotype, among many others.[3].

Infectious disease tests are highly regulated in each country. The International Medical Devices Regulation Forum (IMDF) classifies in vitro diagnostic tests into four categories. The highest risk IVDs include testing for blood-borne pathogens like HIV and hepatitis viruses. In India Central Drugs Standard Control Organisation (CDSCO), MOHFW plays a key central role in the regulation of these devices [4,5].

TAT for infectious serology parameters cannot be compared with clinical chemistry, as the results of the former cannot be released based solely on quality control performance. False positivity due to interference from extremely high titers of antibodies to immunological components, such as streptavidin, can occur. False-negative results have also been reported, but this may be attributed to the assay's limited spectrum for mutants circulating in the region. To overcome these limitations, reporting based on two test strategies is also experimented with, which increases TAT. Therefore, integrating services and subsequent auto-verification remain a challenge.

Testing of infectious serology by CLIA increases throughput, but its sensitivity decreases at low S/CO values, necessitating confirmatory testing. Therefore, adding other specific testing strategies like rapid immunochromatography to S/CO bases algorithms improves the quality of reporting, which needs to be additionally incorporated in the workflow of the TLA laboratory. In this regard, several reports have been published where final reporting is done based on two test strategies. Even national guidelines for diagnostic testing of HIV substantiate this strategy [2].

As with any automation opportunity, it comes with challenges. Automation and artificial intelligence have helped laboratories increase throughput and efficiency. Total laboratory automation is the present of many laboratories and the future of many. Infectious serology, being part of the scope of services provided in TLA, is the pragmatic approach since both sample and equipment are one. But, quality control of tests and the unavailability of uniform standards remain a challenge that requires practical, urgent, and innovative solutions. Until then, the pragmatic approach of infection serology in TLA and appropriate quality control in TLA setting has a long parallel path, and an intersection is debatable.
